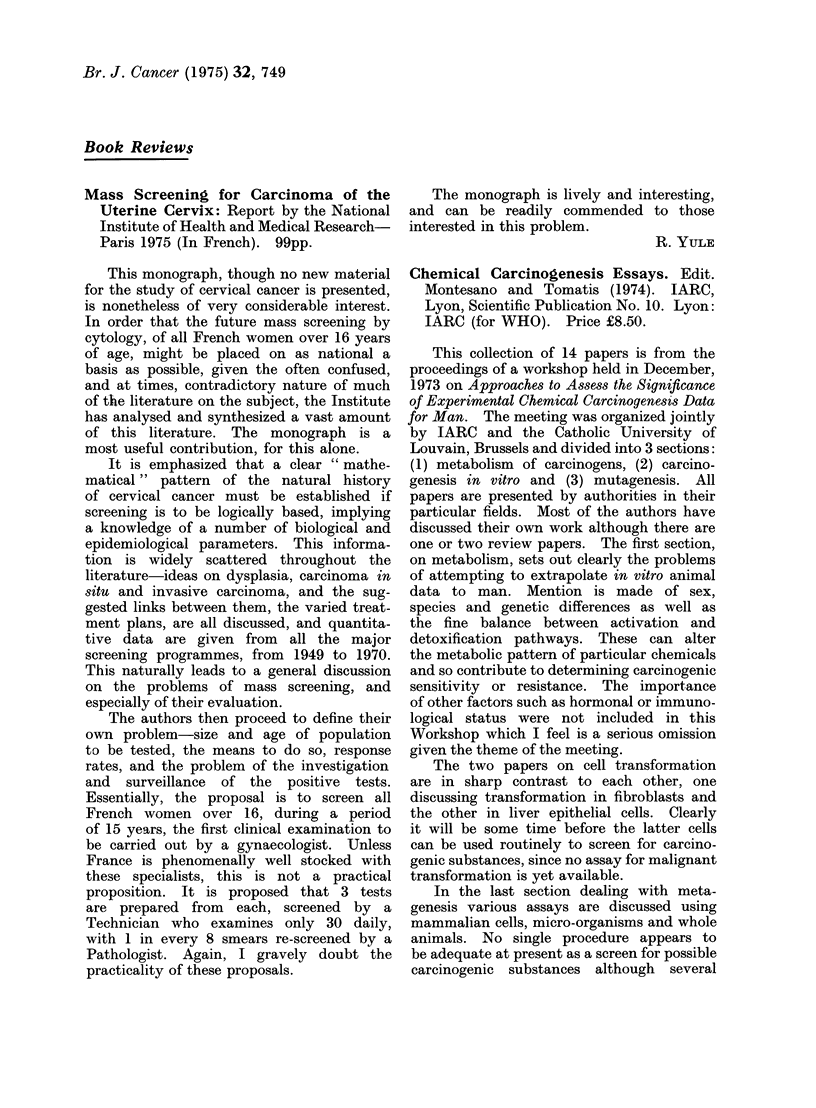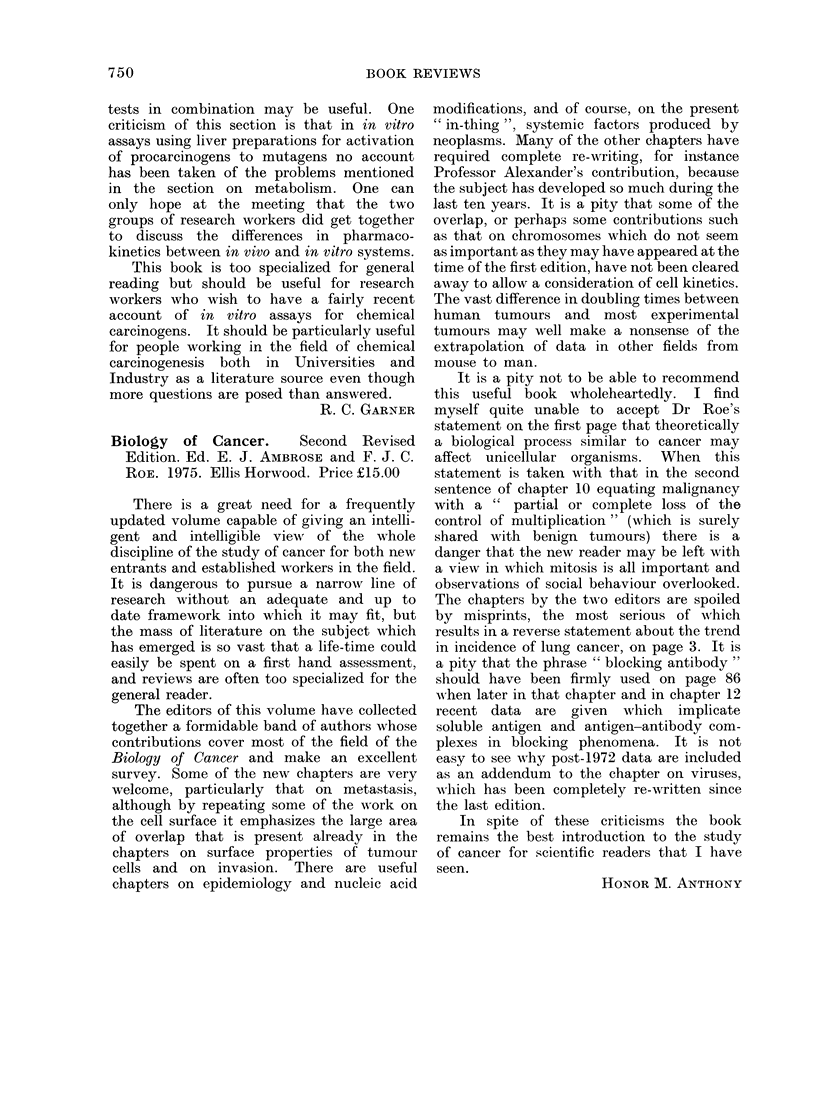# Chemical Carcinogenesis Essays

**Published:** 1975-12

**Authors:** R. C. Garner


**Chemical Carcinogenesis Essays.** Edit. Montesano and Tomatis (1974). IARC, Lyon, Scientific Publication No. 10. Lyon: IARC (for WHO). Price £8.50.

This collection of 14 papers is from the proceedings of a workshop held in December, 1973 on *Approaches to Assess the Significance of Experimental Chemical Carcinogenesis Data for Man.* The meeting was organized jointly by IARC and the Catholic University of Louvain, Brussels and divided into 3 sections: (1) metabolism of carcinogens, (2) carcinogenesis *in vitro* and (3) mutagenesis. All papers are presented by authorities in their particular fields. Most of the authors have discussed their own work although there are one or two review papers. The first section, on metabolism, sets out clearly the problems of attempting to extrapolate *in vitro* animal data to man. Mention is made of sex, species and genetic differences as well as the fine balance between activation and detoxification pathways. These can alter the metabolic pattern of particular chemicals and so contribute to determining carcinogenic sensitivity or resistance. The importance of other factors such as hormonal or immunological status were not included in this Workshop which I feel is a serious omission given the theme of the meeting.

The two papers on cell transformation are in sharp contrast to each other, one discussing transformation in fibroblasts and the other in liver epithelial cells. Clearly it will be some time before the latter cells can be used routinely to screen for carcinogenic substances, since no assay for malignant transformation is yet available.

In the last section dealing with metagenesis various assays are discussed using mammalian cells, micro-organisms and whole animals. No single procedure appears to be adequate at present as a screen for possible carcinogenic substances although several tests in combination may be useful. One criticism of this section is that in *in vitro* assays using liver preparations for activation of procarcinogens to mutagens no account has been taken of the problems mentioned in the section on metabolism. One can only hope at the meeting that the two groups of research workers did get together to discuss the differences in pharmacokinetics between *in vivo* and *in vitro* systems.

This book is too specialized for general reading but should be useful for research workers who wish to have a fairly recent account of *in vitro* assays for chemical carcinogens. It should be particularly useful for people working in the field of chemical carcinogenesis both in Universities and Industry as a literature source even though more questions are posed than answered.

R. C. Garner